# Supplementary material for: Identification and functional analysis of non-coding regulatory small RNA FenSr3 in Bacillus amyloliquefaciens LPB-18
Source: PeerJ. 2023 May 15;11:e15236. doi: 10.7717/peerj.15236 (PMC10194069; doi:10.7717/peerj.15236)
Supplement: Supplemental Information 4 [file peerj-11-15236-s004.zip › KO/CK-vs-T1_map/map03460.html]

KEGG PATHWAY: Fanconi anemia pathway - Reference pathway


|  |  |
| --- | --- |
| **Fanconi anemia pathway - Reference pathway** |  |

[
Pathway menu
| Organism menu
| Pathway entry
| Show description
| User data mapping
]

The Fanconi anemia pathway is required for the efficient repair of damaged DNA, especially interstrand cross-links (ICLs). DNA ICL is directly recognized by FANCM and associated proteins, that recruit the FA core complex. The FA core complex monoubiquitinates FANCD2 and FANCI. The monoubiquitinated FANCD2/FANCI becomes an active form and interacts with a series of DNA repair proteins and facilitates downstream repair pathways. Fanconi anemia is caused by mutations in one of at least 13 FA genes and is characterized by congenital growth abnormalities, bone marrow failure and cancer predisposition.

|  |  |  |
| --- | --- | --- |
| Reference pathway | 184% 150% 122% 100% 82% 67% 55% | 图片下载 |
